# Consciousness transitions during epilepsy seizures through the lens of integrated information theory

**DOI:** 10.1038/s41598-024-56045-x

**Published:** 2024-03-04

**Authors:** F. H. Baglivo, N. Campora, C. J. Mininni, S. Kochen, S. Lew

**Affiliations:** 1 Universidad de Buenos Aires, Instituto de Ingeniería Biomédica, Buenos Aires, Argentina; 2 Estudios en Neurociencias y Sistemas Complejos, CONICET, Buenos Aires, Argentina; 3 Instituto de Biología y Medicina Experimental, CONICET, Buenos Aires, Argentina

**Keywords:** Integrated information, Consciousness, Epilepsy, Diseases of the nervous system, Neural circuits

## Abstract

Consciousness is one of the most complex aspects of human experience. Studying the mechanisms involved in the transitions among different levels of consciousness remains as one of the greatest challenges in neuroscience. In this study we use a measure of integrated information (Φ_AR_) to evaluate dynamic changes during consciousness transitions. We applied the measure to intracranial electroencephalography (SEEG) recordings collected from 6 patients that suffer from refractory epilepsy, taking into account inter-ictal, pre-ictal and ictal periods. We analyzed the dynamical evolution of Φ_AR_ in groups of electrode contacts outside the epileptogenic region and compared it with the Consciousness Seizure Scale (CCS). We show that changes on Φ_AR_ are significantly correlated with changes in the reported states of consciousness.

## Introduction

Consciousness is undoubtedly one of the fundamental aspects, perhaps the most crucial, of human experience. It has been the subject of extensive study for various reasons throughout the history of humanity, captivating the minds of philosophers, physicians, researchers, and other thinkers. Despite centuries of research, it remains a challenging and elusive topic. Having a reliable method for estimating the level of consciousness within a system carries significant implications in diagnostics, as demonstrated by Sitt et al.^1^. In this specific case, they employed event-related potentials, frequency analysis, complexity, and connectivity analysis to investigate the effectiveness of these approaches in distinguishing various states of consciousness among comatose patients. These methods facilitated the examination of individual cases through single-electrode signals and the connectivity between two electrodes. However, they can’t explain complex interactions involving more than two electrodes. In the last decade, a group of researchers has leveraged the advancement of technology and the increasing computational power to conduct experiments and test various theories using image and signal processing. Cutting-edge neuroimaging tools, such as functional magnetic resonance imaging (fMRI), electroencephalography (EEG), and magnetoencephalography (MEG), have allowed them to dive deeper into the neural underpinnings of consciousness. Many theories of consciousness, the Global Workspace Theory (GWT)^2^ and the Integrated Information Theory (IIT)^3^ among them, are being currently tested in different labs across the world. The IIT suggests that consciousness is not a byproduct of processing information in the brain but an intrinsic property that emerges from the integration of information within a system. It also provides measure, the $\Phi$  index, to quantify the level of information integrated within a network, that is, information that cannot be explained as the sum of the individual parts of the system. This index can be estimated from empirical data (i.e. stereoelectroencephalography—SEEG) using an empirical distribution, as proposed by Barret et al.^4^⁠. This measure (or some of its variations) was previously applied to EEG^5^⁠ and fMRI^6^⁠ recordings in humans, and ECoG recordings in monkeys^7^⁠ but it hasn’t been computed on SEEG data from humans.

In this study, we examined transitions in consciousness level during epileptic seizures computing Φ_AR_, a modification of the original proposed integrated information measure Φ^3^, against the Consciousness Seizure Scale (CCS)^8,9^ to determine whether this biomarker could be used as a consciousness level indicator. We also interpreted our results in terms of integration and specialization by means of graph measures, as the mean shortest path and modularity.

## Materials and methods

This study was approved by the Review Ethics Board of El Cruce Hospital, Buenos Aires, Argentina, according to the Declaration of Helsinki. All patients were informed of the purpose and possible consequences of this study and signed an ethical board-approved written informed consent. For this study we analyzed data from 6 adult patients with drug-resistant epilepsy. All of them underwent intracerebral electrodes (SEEG) implantation to determine the precise location of the epileptogenic zone. seizures were those for which we conducted a comprehensive evaluation of consciousness, and the intracerebral signals were relatively free of artifacts.

In this study, we included patients and seizures with a comprehensive evaluation, with a detailed medical record and neurological examination, neuropsychological testing, routine magnetic resonance image (MRI), scalp EEG and SEEG, which resulted in 26 seizures from 6 patients, see Table 1. The SEEG was carried out as part of the patients’ routine clinical care and informed consent was given in the usual way. All seizures were exclusively focal, without progressing to the bilateral tonic-clonic phase. In each patient, seizures exhibited the same ictal onset and propagation.

**Table 1:** Demographic characteristics of the patients included.

Patient ID	Sex/aged (years)	Ictal Semiology	MRI	PET	Localization of deep electrodes	Number of electrodes	Number of contact	Number of seizures included	Epileptogenic zone	Laterality	Engel
Patient 1	M/37	Alteration of consciousness and immobility, oral and right hand automatisms	Normal	Right temporal and left parieto-occipital hypoperfusion	Head, Body and Tail of Hippocampus, bilaterally	6	42	4	Mesial temporal	Left	II
Patient 2	M/20	Deja vu,alteration of consciousness, immobility and swallowing automatisms	Normal	Left temporal hypometabolism	Amygdala, Head and Body of Hippocampus bilaterally	6	43	5	Mesial temporal	Left/Right	Not surgery yet
Patient 3	M/28	Speech arrest, fixed gaze, alteration of consciousness, immobility, slow right oculocephalic deviation. Some seizures were with secondarily generalized	Frontal cortical dysplasia	Normal	Rectus Gyrus, Anterior and Posterior Cingulum, Head and Body of Hippocampus	5	44	3	Frontal medial	Left	I
Patient 4	F/22	Paraesthesia, bitter taste in the mouth, sharp sound and alteration of consciousness	Normal	Diffuse right frontal cortical hypometabolism	Right Head and Body of hippocampus, amygdala and anterior and posterior insula	5	44	6	Insula	Right	II
Patient 5	M/33	Cephalic and ocular version to the right, deviation of the right labial commissure and aphasia of expression Some seizures were with secondarily generalized	Frontal cortical dysplasia	Unrealized	Inferior-Medial and inferior-posterior frontal gyrus, pre central and post central gyrus, Heschl Gyrus, Head and Body of hippocampus	6	43	4	Frontal lateral	Left	II
Patient 6	M/39	Facial right clonias, arm paresthesias, immobility and complex automatism	Frontal opercular retraction	None	Left head of hippocampus, amygdala, anterior and posterior insula and medial and posterior parietal operculum	7	43	4	Frontal lateral	Left	I

See Fig. S1 for detailed information about electrode implantation.

M, male; F, female; MRI, magnetic resonance image; PET, positron emission tomography.

### Consciousness evaluation

We use the Consciousness Seizure Scale (CSS) for determining the loss of consciousness index (LOC)^8,9^. The scale considers different features of conscious experience, delineating 8 criteria. All items were rated by two different epileptologists (NC and SK): items 1–7 can be scored 0 or 1, while the eighth item from 0 to 2, thus yielding a possible total score of 0–9. In this scale, the higher the score, the more severe is the loss of consciousness. Based on the total score, 3 groups were defined: without LOC (score ≤ 1), an intermediate LOC (score ranging from 2 to 5) and with profound LOC (score ≥ 6).

### Electrode’s implantation

Depth electrodes (Ad Tech) had: (a) 8 or 10 platinum contacts with 5- or 10-mm inter-contact center to center distance, contact length of 2.4 mm and 1.1 mm diameter, or (b) 9 platinum contacts with 3 mm distance between the first and the second contact and 6 mm inter-electrode distance from the second to the last. Contact length was 1.57 mm and the electrode diameter was 1.28 mm. Electrodes were identified by a letter of the alphabet. There was no standard labeling for each location. Contacts within an electrode were usually identified with numbers beginning from the deepest 0 (contact number 1, corresponding to the tip) to the base. See Fig. S1 for detailed information about electrodes locations.

### Stereoelectroencephalography recordings

SEEG signals were acquired using the software Cervello 1.04.200, sampled at 2000 Hz with bandpass filtering between 0.7 and 200 Hz. The seizure onset was identified by two epileptologists (NC and SK) through independent reviews. The ictal onset was identified as initial SEEG changes, characterized by sustained rhythmic discharges or repetitive spike-wave discharges that cannot be explained by state changes and that resulted in habitual seizure symptoms similar to those reported in previous studies. The seizure onset zone was defined as the contacts where the earlier ictal SEEG changes were seen.

We consider contacts as “compromised” to those in whom, during the visual analysis of the intracerebral activity signal, epileptiform activity is identified at the onset of the seizure or in immediate propagation. Contacts that did not exhibit epileptiform activity were considered as “not compromised”. This classification criteria were consistent for all patients. The average number of compromised contacts was 14 ± 4.

### Data analysis

Let X be a matrix in which rows are the analyzed electrodes and its columns are time steps**.** The method proposes that the effective information generated by a current state X_t_ with respect to the state $\tau$ time-steps ago in the bipartition $\beta$ = {M_1_, M_2_} is defined as the difference between the mutual information generated by the entire system and the sum of the mutual information generated by the parts within the bipartition. Then, the integrated information $\Phi$ is the effective information $\varphi$ of the minimum information partition (MIP) of the system.

Barret and Seth describe in^3^ how to estimate $\Phi$ for gaussian systems using that the mutual information I(X; Y) between two signals X and Y, that can be written as:

1 $$I\left( {X; Y} \right) = H\left( X \right) - H(X|Y)$$

where H is the entropy. If the system is a gaussian one, Eq. (1) can be written as:

2 $$H\left( X \right) = \frac{1}{2}log\left\{ {det\Sigma \left( X \right)} \right\} - \frac{1}{2}n log\left\{ {2\pi e} \right\},$$

3 $$H(X|Y = y) = \frac{1}{2}log\left\{ {det\Sigma \left( {X|Y} \right)} \right\} - \frac{1}{2}n log\left\{ {2\pi e} \right\},$$

4 $$I\left( {X; Y} \right) = \frac{1}{2}log\left\{ {\frac{det\Sigma \left( X \right)}{{det\Sigma (X|Y)}}} \right\}$$

where Σ(X) and Σ(X|Y) are the empirical covariance matrices. Once the mutual information is calculated using these matrices, effective information for a bipartition $\beta$ = {M_1_, M_2_} can be defined as:

5 $$\varphi \left[ {X;\tau ;\beta } \right] = I\left( {X_{t - \tau } , X_{t} } \right) - \mathop \sum \limits_{k = 1}^{2} I\left( {M_{t - \tau }^{k} ,M_{t}^{k} } \right) = \mathop =\frac{1}{2}log\left\{ {\frac{det\Sigma \left( X \right)}{{det\Sigma \left( {X_{t - \tau } |X_{t} } \right)}}} \right\} - \sum \limits_{k = 1}^{2} \frac{1}{2}log\left\{ {\frac{{det\Sigma \left( {M^{k} } \right)}}{{det\Sigma \left( {M_{t - \tau }^{k} |M_{t}^{k} } \right)}}} \right\}$$

Then, the integrated information ${ \Phi }$ can defined as the effective information $\varphi$ for the MIP. That is:

6 $${\Phi }\left[ {X;\tau } \right] = \varphi \left[ {X;\tau ,\beta^{min} \left( \tau \right)} \right]$$

The above calculations are valid only if the data set X comes from a gaussian process, and this is something that can not be granted. For this case, Barret and Seth developed a variation of ${\Phi }$^4^ that can be used without the gaussian assumption. In that case, the partial covariances are replaced by the covariance matrix of the residual E from a linear regression that comes from X _t_ = α + A·X _t−τ_ + E_t_. The residual has zero mean and its covariance matrix is the partial covariance of X _t−τ_ given X _t_ as shown in. With this change $\varphi_{AR}$ can be expressed as:

7 $$\varphi_{AR} \left[ {X;\tau ,\beta } \right] = \frac{1}{2}log\left\{ {\frac{det\Sigma \left( X \right)}{{det\Sigma \left( {E^{X} } \right)}}} \right\} - \mathop \sum \limits_{k = 1}^{2} \frac{1}{2}log\left\{ {\frac{{det\Sigma \left( {M^{k} } \right)}}{{det\Sigma \left( {E^{Mk} } \right)}}} \right\}$$

In this way we obtain an estimation of the integrated information using the empirical covariance matrices skipping the assumption that our data comes from a Gaussian system. Then ${\Phi }_{AR}$ is obtained by looking for the bipartition than minimized $\varphi_{AR}$ divided by a normalization factor:

8 $$L\left( {\left\{ {M^{1} , M^{2} } \right\}} \right) = : \frac{1}{2}log min_{k} \left\{ {\left( {2\pi e} \right)^{{\left| {M^{k} } \right|}} det\Sigma \left( {M^{k} } \right)} \right\}$$

Normalization is necessary because the subsystems could be almost as large as the whole system so they will generate almost as much information as the whole system. Then,

9 $${\Phi }_{AR} \left[ {X; \tau } \right] = : \varphi_{AR} \left[ {X;\tau , \beta^{min} \left( t \right)} \right]$$

where,

10 $$\beta^{min} \left( t \right) = : arg_{\beta } min \left\{ {\frac{{\varphi_{AR} \left[ {X;\tau ,\beta } \right]}}{L\left( \beta \right)}} \right\}$$

Based on the previous explanation, the algorithm presented by Barret & Sett calculates the effective information among all possible configurations of bi-partitions and then finds the MIP to get the value of the index ${\Phi }_{AR}$. We preprocessed the EEG records downsampling it to 200 Hz by taking 1 sample out of 10. We z-scored each channel and then we applied a 50 Hz notch filter to remove line noise. We used a bandpass filter to limit the signal's bandwidth between 4 and 20 Hz. We selected 6 different electrodes from brain regions that were classified as epileptogenic (compromised) and 6 electrodes from regions that were classified as non-epileptogenic (non compromised). The same electrodes were used for all the seizures from the same patient and the selection of these sets of electrodes was based on medical reports. The mean distance between electrodes was calculated and compared for each group of electrodes (epileptogenic vs non-epileptogenic) without finding statistical differences. We then used Barret’s scripts^4^ to calculate Φ_AR_. A 200 samples (1 s) sliding window, with 100 samples (500 ms) overlapping and $\tau$ = 50 samples (250 ms) was used to generate the data matrices X employed to compute Φ_AR_.

Finally, we computed ΔΦ_AR_ computing the minimum Φ_AR_ inside a 1 s window taken right after the seizure onset and subtracting a baseline value computed as the average of a 2.5 s window located 10 min before the seizure onset. We used the same approach to compute the signal power differences (ΔP) between pre ictal and ictal conditions.

It has been shown that integrated information is maximized when a system is functionally integrated and specialized^9,10^⁠. A reduced level of integration or specialization leads to a reduced value of Φ_AR_. To understand the observed Φ_AR_ changes in terms of changes in integration and specialization, we analyzed the interaction between pairs of electrodes. We constructed the matrix R, equal to the absolute value of the correlation matrix, computed on the same time windows as Φ_AR_. For each one of these matrices, we computed graph theory measures over a weight undirected graph with adjacency matrix L = 1−R. In these graphs, nodes (electrodes) were close if their signals were correlated. We computed two graph-theoretic measures: the mean shortest path length (MSP) and the modularity (Q). The MSP is obtained by finding the minimum distance between each pair of nodes and taking their average. Modularity is defined as the average weight within communities minus the expected average weight for a random graph of equal in-degree and out-degree. Community partitioning was performed with the spectral method of Leich et al.^11^⁠. Both measures were computed with the “Brain Connectivity Toolbox”^12^⁠.

## Results

In this study we analyzed SEEG recordings from 6 epilepsy patients (2 temporal and 4 frontal), candidates to surgery. All the subjects underwent presurgical evaluation at Hospital El Cruce “Nestor Kirchner”, Florencio Varela, Argentina, between 2012 and 2017. The mean age was 30 ± 6.86 years-old, the mean age at first seizure was 12.5± 6.5 years-old and 5/6 were males. The number of implanted electrodes was between 5 and 7 per patient.

To ascertain the continued validity of the index within brain regions severely affected by epileptic seizures, we selected electrodes from both epileptogenic and non-epileptogenic regions, based on physicians reports, conforming two distinct groups: compromised and non-compromised.

**Figure 1:**
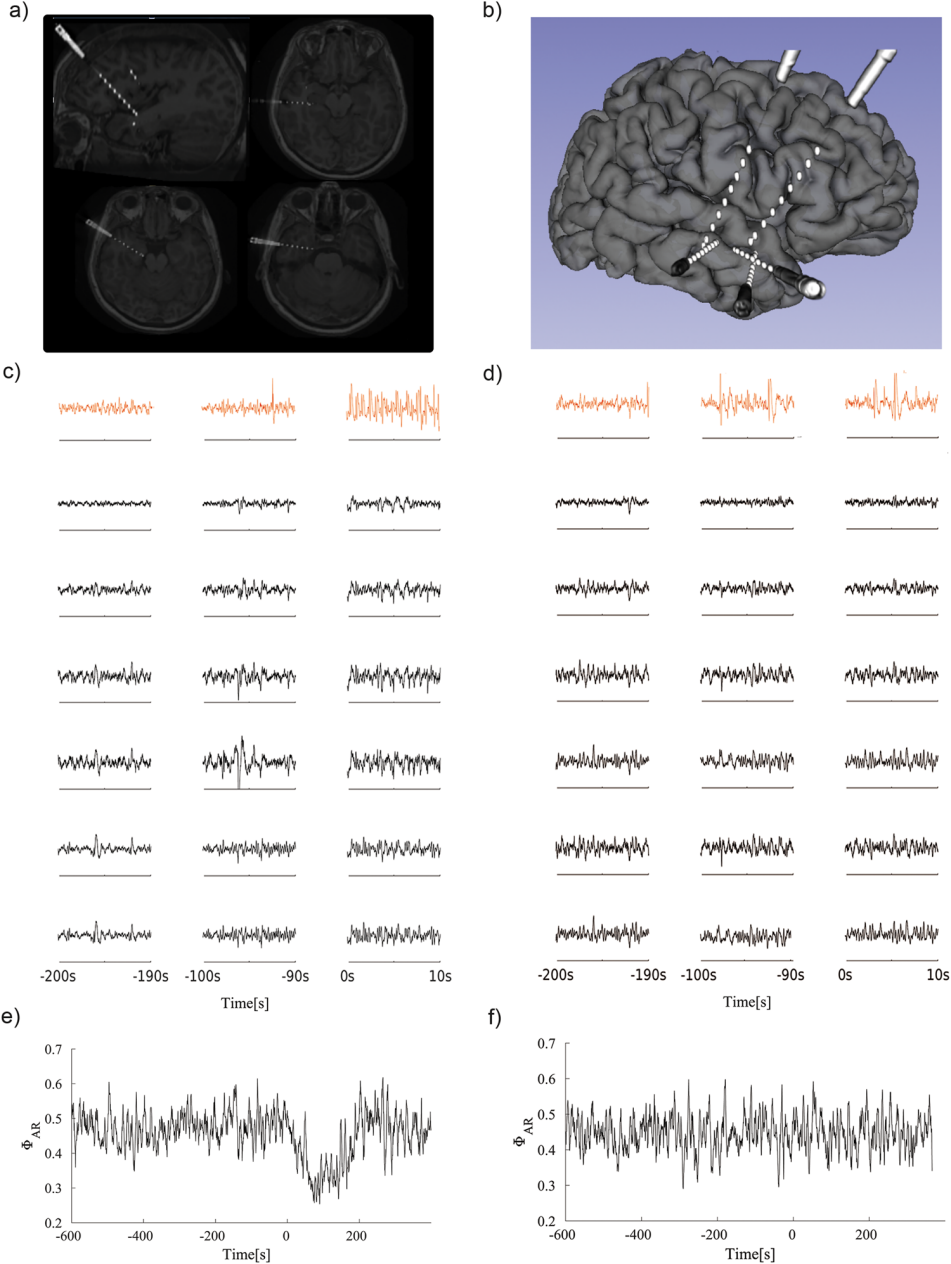
(**a**) Fusion of the pre-surgery magnetic resonance (MR) and post electrode implantation cerebral tomography (CT) image. (**b**) Post implantation three-dimensional brain reconstruction built from pre-implantation MR and post-implantation computed tomography (CT). (**c**) SEEG recording of one electrode in the compromised region (red) and six electrodes in the non-compromised region for a CSS value of 6. (**d**) SEEG recording of one electrode in the compromised zone (red) and six electrodes in the non compromised zone for a CSS value of 0. (**e**) Φ_AR_ value for the 6 SEEG non compromised region recordings in (**c**). (**f**) Φ_AR_ value for the 6 SEEG non compromised region recordings in (**d**).

A typical case is shown in Fig. 1a and b, where 5 macroelectrodes were implanted in the right amygdala, hippocampus, and insula. Windows of SEEG recordings lasting ten seconds are displayed in Fig. 1c and d, showing the activity of a compromised electrode (capturing epileptogenic activity in red) and the activity of six non-compromised electrodes (in black). The signals in Fig. 1c and d come from two different seizures of the same patient. The main difference between them is that the seizure in Fig. 1c is associated with a CSS value of 6 (indicating consciousness loss), while the seizure in Fig. 1d has a value of 0 (no consciousness loss). Figure 1e and f present the values of Φ_AR_ computed over the group of non-compromised electrodes in Fig. 1c and d throughout the entire recording (see “Materials and methods”). Notably, Φ_AR_ exhibits an abrupt and sustained descent at the time of seizure onset only in the seizure where a deep loss of consciousness was reported.

**Figure 2:**
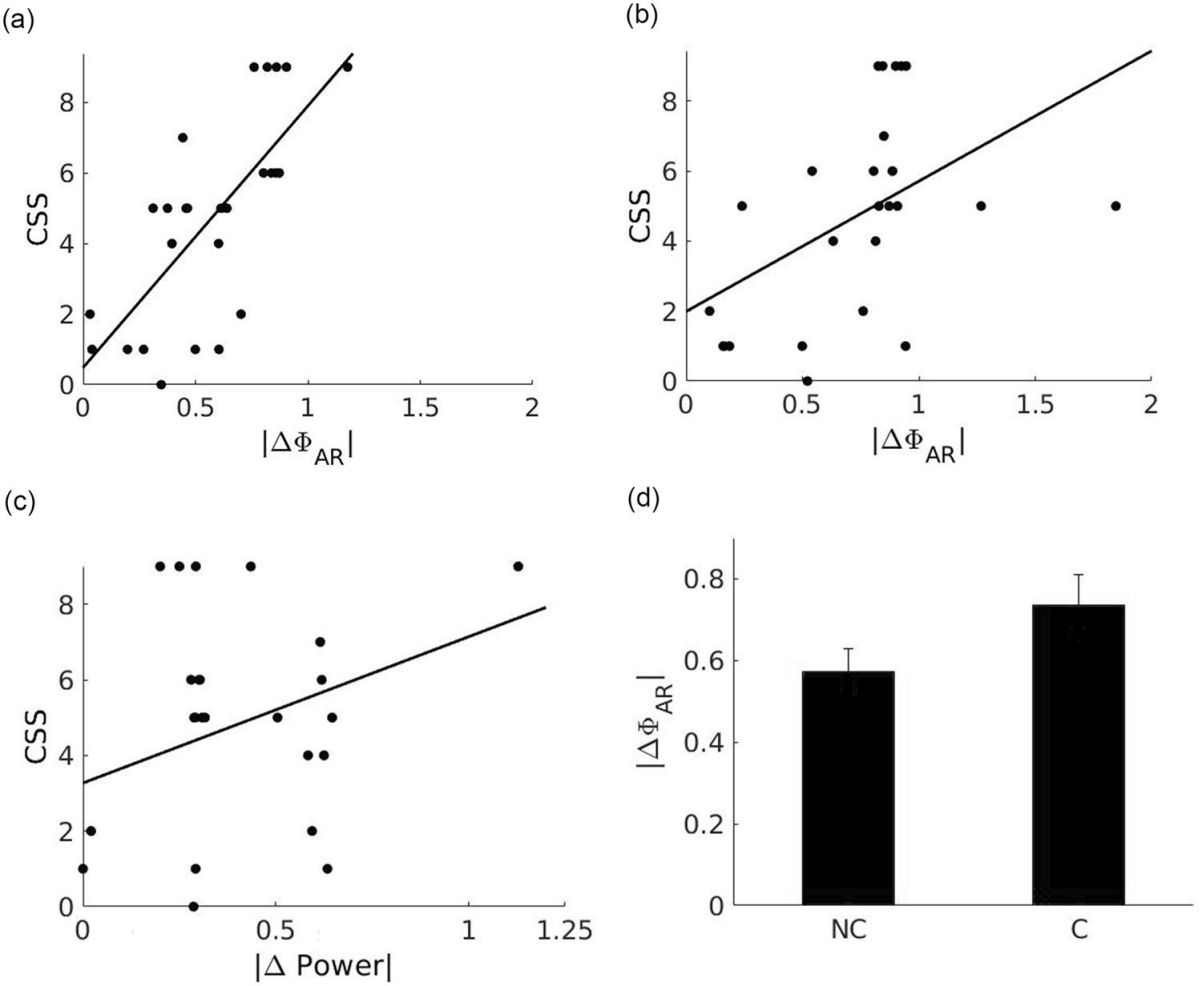
(**a**) Scatter plot of |ΔΦ_AR_| versus CSS for each recording in the non compromised regions. The Pearson correlation coefficient is 0.78 with p < 5e-05 and linear fit equal to 7.38x—0.43. (**b**) Scatter plot of |ΔΦ_AR_| versus CSS for each recording inside the compromised regions. The Pearson correlation coefficient is 0.4593, with p = 0.0275 and linear fit equals to 3.25*x + 2.22, (**c**) Scatter plot of ΔPower versus CSS for each recording in the non compromised zone. The correlation value is 0.37 with p = 0.08 and correlation function equal to 3.88*x + 3.14, (**d**) |ΔΦ_AR_| average value for compromised (0.73) and non compromised groups (0.57) comparison with p < 5e-09.

When this phenomenon was analyzed across all patients and seizures, we found a correlation between the magnitude of the change in Φ_AR_ (ΔΦ_AR_) and the CSS index for both, non-compromised and compromised electrodes (Fig. 2a and b, respectively). We next investigated whether the observed changes in ΔΦ_AR_ could be attributed to changes in the signal power. Although some functional dependence can be observed as in other studies^13,14^, it is not strong enough to conclude that the changes in signal power are solely responsible for the observed changes in ΔΦ_AR_ (Fig. 2c).

Interestingly, we observed that despite the changes in Φ_AR_ being larger for compromised electrodes compared to non-compromised ones (Fig. 2d), the correlation between ΔΦ_AR_ and CSS value for compromised regions was lower, mainly due to the fact that in the epileptogenic zone ΔΦ_AR_ is exacerbated, as shown in Fig. 2b.

**Figure 3:**
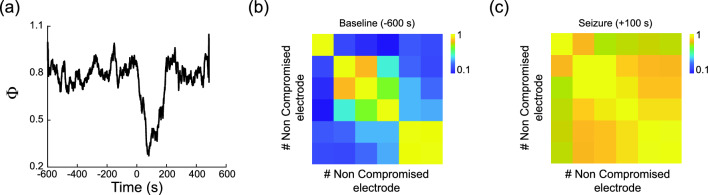
(**a**) Φ_AR_ value for a high CSS value non compromised zone record. (**b**) Electrodes correlation matrix for a 6 electrodes group during baseline (− 600 s). (**c**) Electrodes correlation matrix for a six-electrodes group 100 s after seizure. Bars indicate measured correlation limits, heatmaps were created by using matplotlib 3.8.2 for python 3.

According to the integration information theory of consciousness, the consciousness level is high when brain regions integrate information without losing their specialized functions. We therefore sought to explain the observed relationship between Φ_AR_ and CSS in terms of the observed integration and specialization in compromised and non-compromised electrodes. To this end we first computed the matrix R, the absolute correlation matrix between pairs of electrodes (one matrix for compromised, and one for non-compromised electrodes, for each time window). We show in Fig. 3a the Φ_AR_ measured in one non-compromised electrode during a seizure, together with matrix R before (Fig. 3b) and during (Fig. 3c) the seizure. Then, for each matrix R we obtained matrix L = 1−R, which we employed to define an undirected weighted graph. Each node in this graph stands for an electrode, and pairs of nodes are separated by a distance which is inversely proportional to their absolute correlation. We computed two graph-theoretical measures over this graph: mean shortest path, which quantifies the degree of coupling among recorded areas (a measure of integration), and modularity, which quantifies to what degree the graph can be clustered into groups of nodes characterized by being close to each other, and far from the others (a measure of specialization). Here, a lower mean shortest path is expected for high integration, and a low modularity is expected for low specialization.

**Figure 4:**
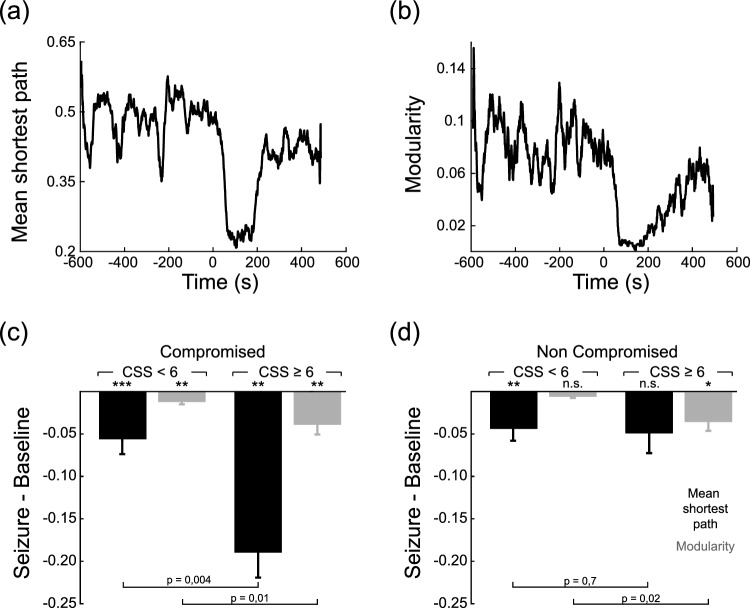
(**a**) Mean shortest path and (**b**) modularity index for the same case as in Fig. 3. (**c**) Mean shorter path and modularity comparison when grouping by CSS < 6 and CSS ≥ 6 for electrodes located in the compromised region. For the first case, the averaged mean shorter path value for the group is − 0.056 ± 0.017 and modularity is − 0.012 ± 0.003. For the second group the averaged mean shorter path value for the group is − 0.19 ± 0.03 and modularity is − 0.039 ± 0.012. (**d**) Mean shorter path and modularity comparison when grouping by CSS < 6 and CSS ≥ 6 for electrodes located in the non compromised region. For the first case, the averaged mean shorter path value for the group is − 0.044 ± 0.014 and modularity is − 0.0062 ± 0.0015. For the second group the averaged mean shorter path value for the group is − 0.049 ± 0.02 and modularity is − 0.036 ± 0.011. (Crisis-basal significance with sign test (*: p < 0.05, **: p < 0.01, ***: p < 0.001). Comparison between CSS groups with Wilcoxon rank-sum test).

We observed a decrease in both mean shortest path and modularity after seizure onset (Fig. 4a and b), pointing to an excess of integration together with a loss of specialization during the seizure. Compromised and non-compromised electrodes showed a qualitative similar picture, but differences appear when associated CSS values are taken into account (Fig. 4c and d). The drop in mean shortest path in compromised electrodes was significantly higher in seizures with profound loss of consciousness, in relation to seizures with moderate loss of consciousness (Fig. 4c). On the other hand, non compromised electrodes showed no significant differences in mean shortest path, and a higher drop in modularity during seizures with profound loss of consciousness (Fig. 4d). These results show that the link between consciousness level and changes in Φ_AR_ can be explained in terms of an increment in integration in parallel with a loss in specialization during the epileptic seizure. Moreover, they point towards integration as the critical factor behind the higher variance explained by non-compromised electrodes in the relationship between Φ_AR_ and CSS (Fig. 2a and b).

## Discussion

Consciousness is a fundamental characteristic of human experience and is closely linked to our ability to perceive, think, reason, and have subjective experiences. It has been studied for centuries by philosophers, scientists, physicians, among others, due to its relevance in understanding human behavior, self-perception, and mental disorders. In this paper, we had a unique opportunity to study it by accessing intracranial EEG (SEEG) records and investigate consciousness transitions in brain regions affected by acute epilepsy seizures and some other non-epileptogenic regions. For this we selected the Integrated Information Theory (IIT)^3,9^⁠. It is based on the idea that consciousness is not simply an emergent property of information processing, but rather it is a property related to the integration of information within a system. In short, and at the fundamental level, consciousness is integrated information and, in his own words, “the integrated information is the amount of information generated by a complex of elements, above and beyond the information generated by its parts”^15^.

After computing Φ_AR_ and its correlation with the CSS index, we observed that the |ΔΦ_AR_| index better explains the variance in the CSS index when the selected electrodes were not within the epileptogenic region, even though it is, on average, greater within the epileptogenic regions. To rule out the possibility that these results could be explained by differences in signal amplitude during seizures, we calculated the differences in signal power (ΔP) and correlated it with the CSS value. We found that the correlation is low and not statistically significant. This indicates that the observed correlation between |ΔΦ_AR_| and the CSS value is not solely influenced by differences in signal amplitude during seizures. Our results are consistent with those presented by Dong et al.^5^⁠. In their study, the authors calculated the Φ index using an EEG signals database obtained from patients who were sedated using different doses of two types of sedatives, resulting in varying levels of consciousness. Their findings indicated that the alpha band Φ tended to differentiate states of consciousness. Furthermore, they demonstrated that the Φ index value decreases as sedation becomes more profound. Similar results were found by Nemirovsky et al.^6^⁠ using fMRI records from 17 patients who were also sedated with different doses. They computed μ[Φmax], a weighted average of the Φ index over the time-series of different networks. The authors showed that the index is sensitive to the different doses in the fronto-parietal network (FPN), a network that is associated with consciousness. The reduction in signal complexity, as noted in Φ, is not unique to this metric; it is also evident in various other complexity measures. El Youssef et al.^16^ employed permutation entropy in their analysis of SEEG recordings. Their findings revealed a decline in signal complexity during seizures, providing insight into the manner in which the loss of signal complexity spreads within the cortex, correlating with alterations in awareness.

In terms of graph theory, we found a sudden decrease in the mean shortest path in the compromised regions for seizures with profound loss of consciousness. This suggests that this change in Φ_AR_ with respect to the baseline could be attributed to high integration processes occurring in those epileptogenic regions. In contrast, for regions outside the epileptogenic areas, we observed a decrease in modularity, indicating a loss of specialization during profound loss of consciousness, which causes a decrease in Φ_AR_. These findings suggest that, during the seizures, the level of specialization decreases outside the epileptogenic region, and this in turn leads to lower integrated information and a concomitant loss of consciousness, as evidenced through the CSS values.

Numerous research groups have recently tested key theories of consciousness, including Integrated Information Theory (IIT)^17^. However, the outcomes have not yielded conclusive results, indicating a need for further refinement of these theories. In this context, we posit that Φ, as a metric aligned with the postulates of IIT, offers distinct advantages over alternative theories. One notable advantage is its consideration of consciousness as a unique and irreducible phenomenon, a characteristic absent in individuals who have undergone cerebral commissure sectioning^18^, which report non-unique conscious experiences when exposed to compound stimuli. It is crucial to emphasize that Φ can incorporate as many sources (contacts) as desired, making it a global dependence measure. This versatility allows for a comprehensive evaluation of interactions across a wide array of sources, contributing to its significance as a metric for assessing global dependencies in the context of consciousness studies.

Consciousness is thought to involve many brain regions, and IIT in particular assumes that the whole brain should be considered in order to have a good estimate of the consciousness level. Since SEEG recordings, by their own nature, preclude an even representation of the whole brain, our measurements can only be a lower bound on the “true” Φ values. Nonetheless, our results show that, even when computed on brain signals from relatively close brain regions, Φ captures information that correlates with the awareness state of the subjects. Since compromised electrodes are usually close to the epileptogenic zone, and non compromised electrodes are usually far from it, it can be hypothesized that it is the distance from the epileptogenic zone the variable that makes a brain region more informative about the consciousness state. The epileptogenic region itself would be less informative, since it is involved in all seizures, regardless of the conscientiousness level. The regions far from the epileptogenic zone could be more or less recruited, and this would correlate with consciousness level. However, how this propagation is measured is not trivial. For instance, the CSS measured in our data did not correlate with signal power in non compromised electrodes, while changes in Φ value did. The uneven distribution of brain regions sampled could be compensated by considering more subjects with contacts in other brain regions, and by developing new methods capable of measuring how much each separate brain region contributes to the global Φ.

## Conclusions

Our results, which are novel as they stem from SEEG signals, provide further support for the sensitivity of Φ_AR_ to shifts in consciousness. To enhance our explanatory capacity, we conclude that the inclusion of mean shortest path and modularity indexes augments the outcomes derived from Φ_AR_. This additional insight aids in comprehending the underlying sources of its fluctuations. Although our results are in agreement with the IIT at a population level, more data is needed to confirm that Φ_AR_ is a reliable marker of consciousness alteration on a patient-by-patient basis.

### Supplementary Information

Supplementary Figure S1.

## Data Availability

The data that support the findings of this study are available from “Hospital El Cruce”, Buenos Aires, Argentina, but restrictions apply to the availability of these data, which were used under license for the current study, and so are not publicly available. Data are however available from the authors upon reasonable request to Dr. Silvia Kochen (skochen@gmail.com) and with permission of “Hospital El Cruce”, Buenos Aires, Argentina.
