# Supplementary material for: Consciousness transitions during epilepsy seizures through the lens of integrated information theory
Source: Sci Rep. 2024 Mar 4;14:5355. doi: 10.1038/s41598-024-56045-x (PMC10912751; doi:10.1038/s41598-024-56045-x)
Supplement: Supplementary file 1 — Supplementary Figure S1. [file 41598_2024_56045_MOESM1_ESM.pdf]

Supplementary Information

Consciousness transitions during epilepsy seizures through the lens of Integrated Information Theory

Baglivo, F.H.; Campora, N.; Mininni, C. J., Kochen, S, Lew, S.

A

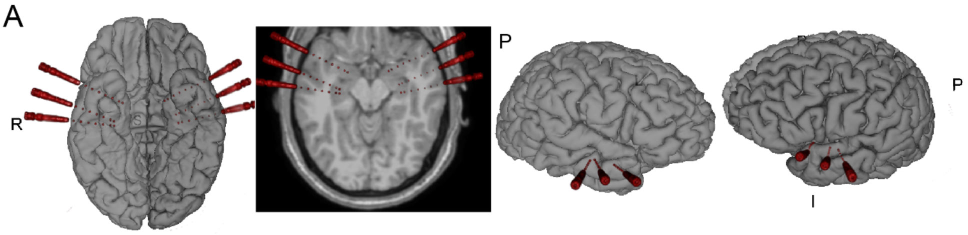

Non compromised contact localization
Superior Temporal Cx
Superior Temporal Cx
Superior Temporal Cx
Medial Temporal Cx
Medial Temporal Cx
Medial Temporal Cx

B

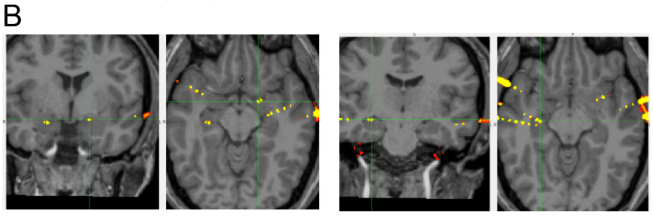

Non compromised contact localization
Superior Temporal Cx
Superior Temporal Cx
Medial Temporal Cx
Medial Temporal Cx
Medial Temporal Cx
Medial Temporal Cx

C

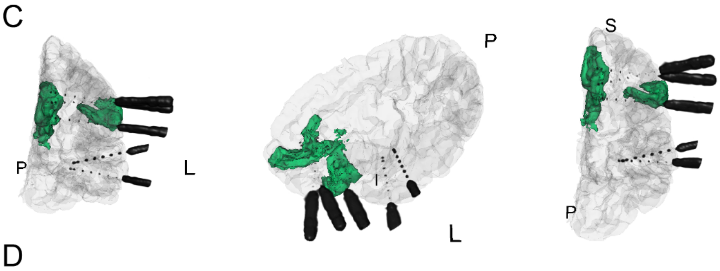

Non compromised contact localization
Medial Temporal Cx
Medial Temporal Cx
Medial Temporal Cx
Medial Temporal Cx
Medial Temporal Cx
Medial Temporal Cx

D

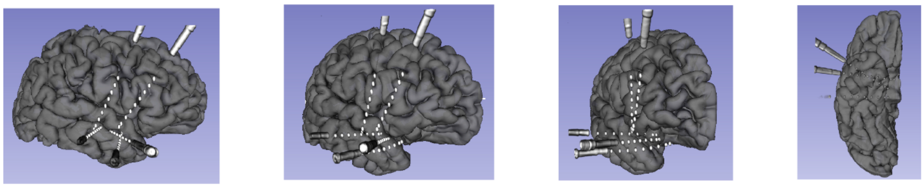

Non compromised contact localization
Enthorinal Cx
Hippocampus
Hippocampus
Hippocampus
Hippocampus
Hippocampus

E

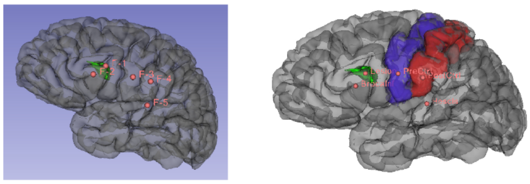

Non compromised contact localization
Insula
Medial Temporal Cx
Medial Temporal Cx
Medial Temporal Cx
Medial Temporal Cx
Medial Temporal Cx

F

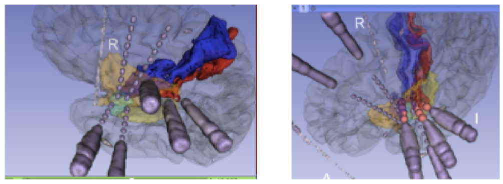

Non compromised contact localization
Left Hippocampus
Left Hippocampus
Left parahippocampal gyrus
Left Amygdala
Left Insula
SuperiosTemporal Gyrus

Figure S1. Left) Post implantation three-dimensional brain reconstruction built from pre-implantation MR and post-implantation computed tomography (CT). Figures A to F belong to Patients 1 to 6 in Table 1. Right) Non compromised contact localization for each patient. These were the contacts employed to compute the measure across all seizures for each patient.
